# Peripheral Blood T Cell Gene Expression Responses to Exercise and HMB in Sarcopenia

**DOI:** 10.3390/nu13072313

**Published:** 2021-07-05

**Authors:** Suk-Ling Ma, Junyi Wu, Liuying Zhu, Ruth Suk-Mei Chan, Xingyan Wang, Dan Huang, Nelson Leung-Sang Tang, Jean Woo

**Affiliations:** 1 Department of Psychiatry, The Chinese University of Hong Kong, Hong Kong, China; suklingma@cuhk.edu.hk; 2 Department of Chemical Pathology, The Chinese University of Hong Kong, Hong Kong, China; wujunyi1688@163.com (J.W.); beya_002@163.com (X.W.); 3 Department of Medicine and Therapeutics, The Chinese University of Hong Kong, Hong Kong, China; zoezhuliuying@link.cuhk.edu.hk (L.Z.); jeanwoowong@cuhk.edu.hk (J.W.); 4 Department of Applied Biology and Chemical Technology, The Hong Kong Polytechnic University, Hong Kong, China; ruth.chansm@gmail.com; 5 Cytomics Limited, Building 19W, Hong Kong Science Park, Hong Kong, China; danhuang2018dana@gmail.com; 6 Hong Kong Branch of CAS Center for Excellence in Animal Evolution and Genetics, Hong Kong, China; 7 Functional Genomics and Biostatistical Computing Laboratory, CUHK Shenzhen Research Institute, Shenzhen 518172, China

**Keywords:** sarcopenia, exercise, nutrient supplementation, gene expression

## Abstract

Background: Sarcopenia is a major health problem in older adults. Exercise and nutrient supplementation have been shown to be effective interventions but there are limited studies to investigate their effects on the management of sarcopenia and its possible underlying mechanisms. Here, we studied T cell gene expression responses to interventions in sarcopenia. Methods: The results of this study were part of a completed trial examining the effectiveness of a 12-week intervention with exercise and nutrition supplementation in community-dwelling Chinese older adults with sarcopenia, based on the available blood samples at baseline and 12 weeks from 46 randomized participants from three study groups, namely: exercise program alone (*n* = 11), combined-exercise program and nutrition supplement (*n* = 23), and waitlist control group (*n* = 12). T cell gene expression was evaluated, with emphasis on inflammation-related genes. Real-time PCR (RT-PCR) was performed on CD3 T cells in 38 selected genes. Correlation analysis was performed to relate the results of gene expression analysis with lower limb muscle strength performance, measured using leg extension tests. Results: Our results showed a significant improvement in leg extension for both the exercise program alone and the combined groups (*p* < 0.001). Nine genes showed significant pre- and post-difference in gene expression over 12 weeks of intervention in the combined group. Seven genes (*RASGRP1*, *BIN1*, *LEF1*, *ANXA6*, *IL-7R*, *LRRN3*, and *PRKCQ*) showed an interaction effect between intervention and gene expression levels on leg extension in the confirmatory analysis, with confounder variables controlled and FDR correction. Conclusions: Our findings showed that T cell-specific inflammatory gene expression was changed significantly after 12 weeks of intervention with combined exercise and HMB supplementation in sarcopenia, and that this was associated with lower limb muscle strength performance.

## 1. Introduction

Skeletal muscle comprises about 40% of the body’s mass. Sarcopenia is the progressive loss of skeletal muscle mass and function, resulting from increased catabolism and decreased anabolism of skeletal muscle. It is a major health issue in older adults, affecting 20% of people over the age of 70 and up to 50% of people over 80 [1]. Muscle mass starts to decline from the age of 40, at the rate of 1–2% per year. By the age of 70, up to 30% of muscle mass is lost. Studies have shown that some interventions could reverse sarcopenia, but pharmacological interventions have resulted in limited efficacy [2,3]. Nonpharmacological interventions, including exercise and nutrition supplementation, are more promising prevention measures and intervention strategies for sarcopenia [4].

Reduced physical activity is a risk factor for sarcopenia, and exercise might reverse sarcopenia [3,5]. Resistance training has a significant effect on the improvement of muscle mass, muscle strength, and physical performance [6]. However, due to the heterogeneity in exercise (type, duration, and intensity) performed in different studies, efficacy and persistence varies among studies. Protein intake is important for stimulating muscle protein synthesis. As age increases, there is a significant decline in food intake. Randomized controlled trials and review studies have both shown that exercise increases muscle mass and that this increase is greater when combined with nutrient supplementation [7,8,9]. β-hydroxy-β-methyl-butyrate (HMB) is a metabolite derived from leucine and can be used as a nutrient supplement. It has been shown to modulate muscle protein degradation and promote protein synthesis [10,11]. HMB is widely used by athletes and, in combination with exercise training, can improve the muscle strength. Studies have shown that HMB is effective at improving lean muscle mass and preserving muscle strength in older adults [12]. Combining resistance exercise and HMB supplements increases thigh lean mass [13]. The combination of exercise and HMB has been shown to reduce the level of markers associated with muscle damage and increase the level of aerobic fitness [14]. However, a review study showed controversial results on the effectiveness of combining exercise and HMB for improving muscle strength and performance [7]. Furthermore, most studies on nutrient supplement in the elderly were confined to a poor baseline nutrition status.

In addition to HMB, there are other nutrient supplements, such as vitamin D and omega 3 fatty acid. Vitamin D is important for the optimal functioning of muscle, and vitamin D deficiency is associated with muscle weakness [15]. Vitamin D deficiency is common in older adults and is a risk factor for sarcopenia [16]. Omega 3 fatty acid is known for its anti-inflammatory properties and for promoting muscle protein synthesis. Recent studies have suggested that intaking 0.1–1.2 g/kg/day of protein [17], 800–1000 IU of vitamin D supplementation [18], and 3 g/day of omega 3 supplements might be beneficial for muscle mass and strength [19].

T cells play an active role in many muscle diseases, such as increasing muscle fiber necrosis and muscle cytotoxicity [20]. Recent studies have also shown the importance of the role of T cells in muscle repair. In the context of inflammaging, chronic and low-grade inflammation has been suggested to be associated with various age-related disorders, including sarcopenia, obesity, and coronary heart disease [21]. Whole blood samples capture RNA profiles of all cell types and peripheral blood mononuclear cells (PBMC), composed mainly of granulocytes, platelets, and reticulocytes. The gene expression profile differs significantly with different blood-derived RNA sources [22]. Most studies of this nature have quantified the gene expression level in whole blood samples. However, the expression of each cell subpopulation might be different and quantifying them collectively in whole blood samples might mask the subset-specific response of the intervention, leading to conflicting findings. Therefore, cell-type-specific gene expression is required to give an accurate quantification of gene expression in T cells, regardless of variations in different cell populations.

Our study examined the association between the expression of inflammation-related genes in CD3 T cells and the muscle strength outcome in a sub-sample of older adults with sarcopenia who participated in a randomized controlled trial in order to examine the effectiveness of an intervention with exercise and nutrition supplementation (vitamin D, omega 3 fatty acid, and HMB) for the management of sarcopenia.

## 2. Materials and Methods

### 2.1. Subjects

The results of this study were part of a completed trial examining the effectiveness of an intervention with exercise and nutrition supplementation in older community-dwelling Chinese adults with sarcopenia (ClinicalTrials.gov Identifier: NCT02374268) [23]. In brief, Chinese subjects aged over 65 with sarcopenia were defined with the Asian Working Group criteria [24], and those who fulfilled the eligibility criteria were recruited from the community in Hong Kong. These subjects were randomly assigned to one of the three groups: exercise program alone (exercise), combined-exercise program and nutrition supplementation (combined), or waitlist control group (control). All participants provided written informed consent. The study was performed in compliance with the Declaration of Helsinki and was approved by the Clinical Research Ethics Committee of the Chinese University of Hong Kong.

### 2.2. Intervention

Details of the intervention design have been described previously [23]. In brief, the control group was asked to maintain their usual physical activities and dietary habits during the 6-month study period and were provided with the same exercise program as the other two groups. The exercise group performed 20–30 min resistance exercise and 20 min aerobic exercise on a weekly basis. The combined group received nutrition supplements and performed the same exercise as the exercise group. The nutrition supplements consisted of two sachets of Ensure NutriVigor daily from baseline to 12 weeks. Each sachet (54.1 g powder) contained 231 calories, 8.61 g protein, 1.21 g β-hydroxy β-methylbutyrate, 130 IU vitamin D, and 0.29 g omega-3 fatty acid. The intervention lasted for 12 weeks.

### 2.3. Questionnaires and Measurement

Demographic, lifestyle and medical history data, 3-day dietary record, validated Physical Activity Scale for the Elderly (PASE), and the 12-Item Short Form Health Survey (SF-12) were collected using a standardized questionnaire. Anthropometric measurements, body composition, grip strength, leg extensors strength, muscle power in the upper extremities, physical performances, including five-chair stand test, and usual gait speed were assessed.

### 2.4. Gene Expression

Whole blood samples were collected from subjects by venipuncture. PBMC was isolated using Ficoll gradient centrifugation method (GE Healthcare Corp, Maywood, NJ, USA), and CD3 T cells were isolated by CD3 MicroBeads using magnetic separation. The isolated sample was stabilized by Trizol. Total RNA was extracted by the Trizol-chloroform method, and cDNA was prepared by the standard method. The cDNA transcript was amplified by specific primers for a list of genes that are highly expressed in CD3 T cells, and was quantified using a TB Green Premix Ex Taq II (Tli RNase H Plus) kit (Cat#RR820A, TAKARA) by a LightCycler 480 System (Roche). The relative gene expression level was calculated by a 2-ΔΔCt method using the geometric mean of the CT values of three reference genes (RPS18, RPL31, and B2M) to correct the efficiency of each gene [25].

### 2.5. Statistical Analysis

Differences in variables between baseline and 12 weeks after intervention were analyzed by a linear regression model. The correlation of the variables was analysed by Pearson’s correlation. Interactions of variables were analyzed by linear regression. A nominal *p*-value less than 0.05 was considered a significant association. Multiple comparison correction was corrected using the Benjamini–Hochberg false discovery rate (FDR) procedure [26]. Statistical analysis was performed using SPSS v24.0.

## 3. Results

**Figure 1:**
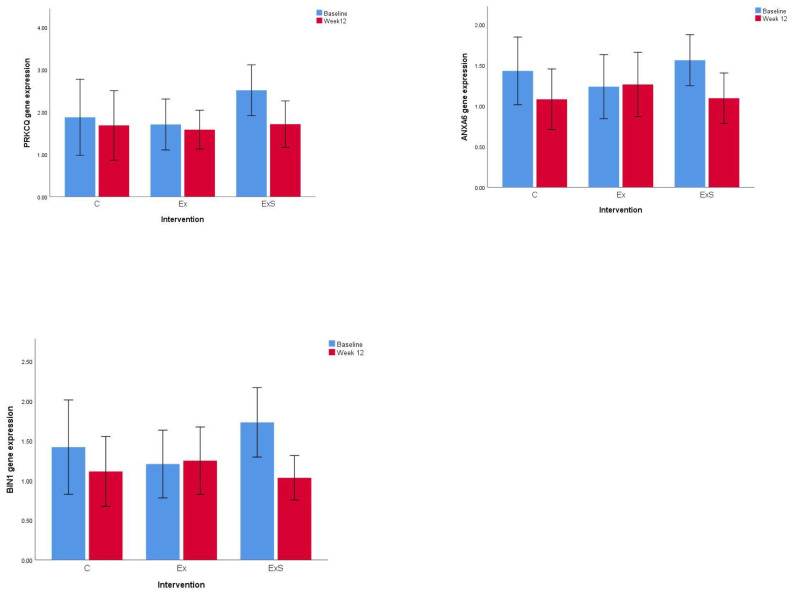
Gene expression levels of three genes (*PRKCQ*, *ANXA6*, and *BIN1*) showing the most significant difference before and after interventions. C: control; Ex: exercise; ExS: exercise and nutrient supplement; The gene expression shown is relative gene expression to geometric mean of 3 house-keeping genes; *p* < 0.05.

Twelve subjects were assigned to the control group, eleven subjects were assigned to the exercise group, and twenty-three subjects were assigned to the combined group. The clinical outcome of the study was described in our previous study [23]. The baseline characteristics of this sub-sample (subjects with blood samples) were similar, except for maximum grip strength and five-chair stand (Table 1). In our previous study, significant changes in lower limb muscle mass, ASM, leg extension, and five-chair stand were observed after 12 weeks of intervention [23]. Therefore, these parameters were investigated in this study. Among the thirty-eight genes we selected for this study, nine genes (*PRKCQ*, *BIN1*, *ANXA6*, *MAF*, *LDHB*, *HINT1*, *SOD1*, *TOMM7*, *EIF3E*) showed significant differences in gene expression between baseline and 12 weeks after combined exercise and nutrient supplement intervention (Figure 1) (Table 2) (Supplementary Table S1). Further analysis was conducted on the interaction effect between interventions and gene expression levels based on the clinical outcome. Seven genes (*RASGRP1*, *BIN1*, *LEF1*, *ANXA6*, *IL-7R*, *LRRN3*, and *PRKCQ*) showed significant interaction between gene expression and intervention among the control, exercise, and combined groups that affected the leg extension (Table 3). There was no significant difference in the gene expression of *RASGRP1*, *BIN1*, *LEF1*, *ANXA6*, *IL-7R*, *LRRN3*, and *PRKCQ* among the three groups at baseline.

**Figure 2:**
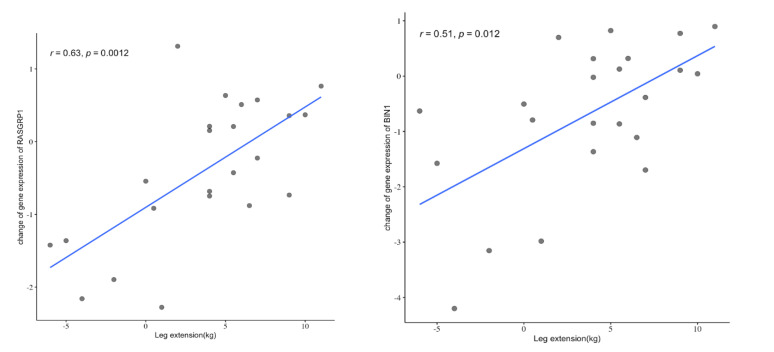

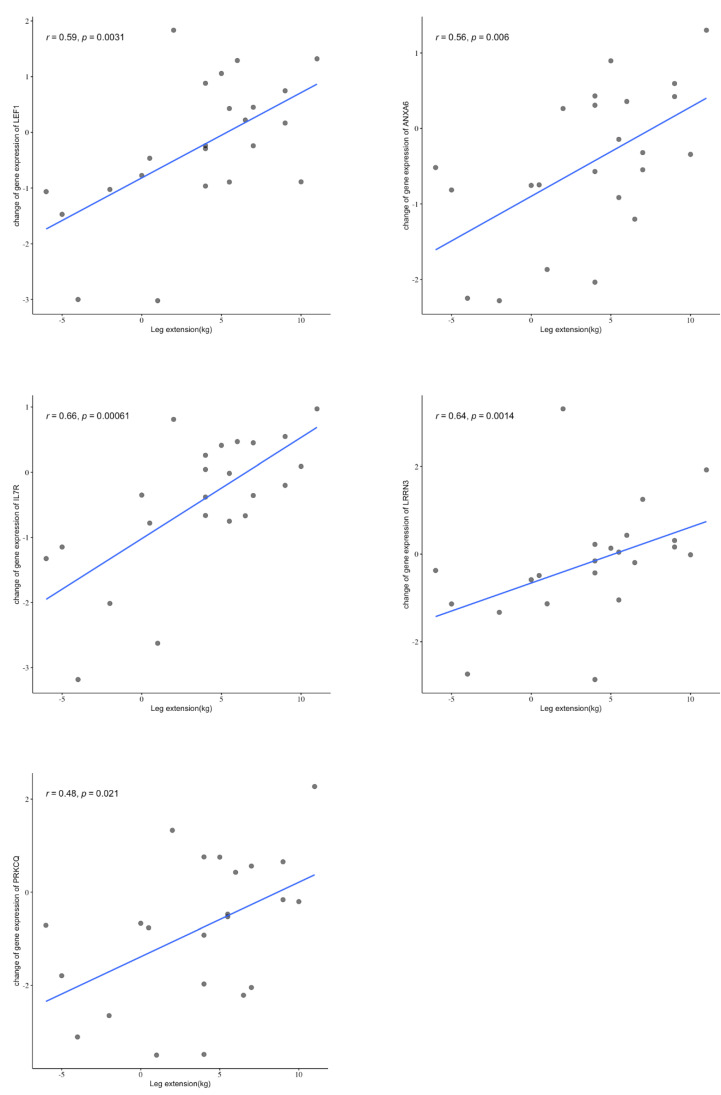
Figure showing the correlation between changes in gene expression and leg extensions in combined group.

We further analyzed the correlation between changes in gene expression and leg extensions and we identified positive correlations between changes in gene expression of *RASGRP1*, *BIN1*, *LEF1*, *ANXA6*, *IL-7R*, *LRRN3*, and *PRKCQ* and leg extensions in the combined group (Table 4) (Figure 2). All six of these genes survived the multiple testing correction and remained statistically significant.

## 4. Discussion

Our previous study showed significant improvements in lower limb muscle mass, ASM, leg extension, and five-chair stand after 12 weeks of intervention [23]. In this study, we aimed to identify the pathways that might be associated with the changes in these parameters. Inflammaging refers to low-grade inflammation, and is suggested to be associated with sarcopenia [21]. In addition, T cells play an important role in muscle regeneration. Therefore, a list of inflammation-related genes expressing in T cells were chosen to compare gene expression levels before and after different interventions. Our results showed that the level of expression of seven genes (*RASGRP1*, *BIN1*, *LEF1*, *ANXA6*, *IL-7R*, *LRRN3*, and *PRKCQ*) was associated with the improvement in leg extensions in the combined exercise and nutrient supplement group. To the best of our knowledge, this is the first study reporting the association of T cell-specific gene expression and intervention in improving leg extensions in subjects with sarcopenia.

In sarcopenia, the balance between muscle protein breakdown and muscle protein synthesis is disturbed. The underlying mechanism for sarcopenia is not well understood, and a number of processes associated with aging have been suggested, including reduction in protein synthesis, dysregulation of proteasomal degradation, mitochondrial dysfunction, increased reactive oxidative stress, and increased inflammation [27]. Exercise and nutrient supplementation are two major interventions for sarcopenia. Cell death and inflammation are involved in aging and may result in reduced muscle mass and strength. On the other hand, exercise can upregulate the anabolism of muscle by increasing protein synthesis. Resistance exercise might stimulate muscle growth in the elderly and may induce a transient redistribution of immune cell populations, which might be associated with an immune response [28].

In recent years, there has been an increasing interest in the potential role of inflammation in sarcopenia. Increased levels of IL-6 and TNF-α and a reduced level of IL-10 have been reported in cases of sarcopenia [29,30]. An imbalance in pro- and anti-inflammatory pathways leads to inflammaging, which is associated with sarcopenia through inhibition of muscle regeneration [31]. T cells have a role in the repair and regeneration of muscle [32]. Impaired muscle regeneration was demonstrated in T cell-deficient mice [33]. More importantly, T cells play an important role in the regulation of the inflammatory response, including the secretion of inflammatory cytokines, such as IL-6 and TNF-α. Our study showed the gene expression level of seven T cell specific inflammatory genes, including *RASGRP1*, *BIN1*, *LEF1*, *ANXA6*, *IL-7R, LRRN3*, and *PRKCQ* was positively correlated with leg extensions. These genes were associated with T cell regulation and inflammation. *RASGRP1* was associated with the regulation of T cell development and differentiation [34]. *LEF1* is a transcription factor essential for the thymocyte maturation [35]. *ANXA6* regulated the proliferation of T cells through IL-2 [36]. IL-7 and *IL-7R* signalling pathways are important for T cell population maintenance and are involved in many inflammatory conditions, such as diabetes and rheumatoid arthritis [37]. PKC-theta (PKCθ) is an enzyme encoded by *PRKCQ* and is a key regulator of signal transduction in T cells [38]. In addition, some of the genes, including *BIN1* and *ANAX6*, were associated with muscle metabolism [39,40]. These findings suggest that gene expression in T cells might play an important role in muscle metabolism and the inflammatory responses associated with the pathogenesis of sarcopenia. Inflammaging suggests that older adults have poorer control of inflammation, a notion that is supported by studies showing that there is a reduced expression of inflammatory-regulating genes, including *LRRN3* and *LEF1*, in the elderly [41]. Our study showed that intervention with exercise and nutrient supplementation in the combined group was associated with changes in expression of seven genes in T cells, which might improve the regulation of inflammation. Reduced inflammation might promote muscle regeneration, and therefore improve the strength of leg extensions.

Our study demonstrated, for the first time, that the expression of some T cell-specific genes is associated with leg extension strength, and that this can be mediated by the combined intervention of exercise and nutrient supplementation. This finding suggests that there is an association between inflammation regulation and muscle regeneration, which could have implications for the development of therapeutic strategies for muscle regeneration. However, this study is limited by its small sample size, and a study with larger sample size is required to further validate the current finding. In addition, future studies investigating the possible mechanism of inflammatory response on muscle regeneration might be useful for drug development.

## Figures and Tables

**Table 1:** Baseline characteristics of the study participants.

	Control (*n* = 12)	Exercise (*n* = 11)	Combined (*n* = 23)
Age, mean (SD)	69.3 (3.8)	76.4 (7.4)	73.7 (6.1)
Female, *n* (%)	6 (50.0)	6 (54.5)	12 (52.2)
Weight, kg, mean (SD)	44.0 (5.4)	47.2 (5.5)	44.6 (6.2)
Height, cm, mean (SD)	151.8 (4.7)	156.9 (6.3)	155.4 (8.0)
Body mass index, kg/m^2^, mean (SD)	19.1 (1.8)	19.1 (1.2)	18.4 (1.6)
CMMSE, mean (SD)	27.0 (3.5)	28.3 (2.4)	28.2 (1.8)
SARC-F, mean (SD)	1.42 (0.80)	1.64 (1.50)	1.77 (2.05)
Maximum grip strength, kg, mean (SD) ^1^	10.63 (5.50)	17.96 (7.41)	13.61 (5.06)
Maximum leg extension, kg, mean (SD)	15.38 (5.67)	17.18 (5.55)	13.37 (5.45)
Five-chair stand test, s, mean (SD) ^1^	9.75 (2.56)	10.07 (3.98)	13.92 (6.28)

^1^ All items were not significantly different between groups, except maximum grip (*p* = 0.015) and five-chair stands (*p* = 0.034). CMMSE, Chinese version Mini-Mental State Exam; SD: standard deviation.

**Table 2:** Table showing genes with significant changes in gene expression before and after combined exercise and nutrient supplement intervention.

Gene	Fold Change	*p*-Value
*PRKCQ*	3.35	0.02
*BIN1*	2.76	0.035
*ANXA6*	1.75	0.023
*MAF*	1.36	0.016
*LDHB*	1.2	0.008
*HINT1*	1.07	0.008
*SOD1*	1.05	0.004
*TOMM7*	0.97	0.014
*EIF3E*	0.87	0.004

**Table 3:** Linear regression analysis showing the interaction of intervention and gene expression in modulating the leg extension result.

Gene	B(SE)	β	T	*p*-Value
*RASGRP1*	1.804 (0.631)	0.350	2.860	0.008
*BIN1*	1.270 (0.482)	0.326	2.637	0.015
*LEF1*	1.095 (0.508)	0.269	2.157	0.037
*ANXA6*	1.642 (0.685)	0.297	2.399	0.012
*IL-7R*	2.166 (0.653)	0.404	3.319	0.003
*LRRN3*	1.137 (0.523)	0.269	2.174	0.024
*PRKCQ*	0.929 (0.422)	0.274	2.200	0.034

B(SE), standard error for the unstandardized beta; β, standardized beta; T, *t* test statistic.

**Table 4:** Correlation analysis between gene expression and leg extension in combined group.

Gene	*r* ^1^	*p*-Value ^2^
*RASGRP1*	0.633	0.001
*BIN1*	0.514	0.012
*LEF1*	0.588	0.003
*ANXA6*	0.555	0.006
*IL-7R*	0.660	0.001
*LRRN3*	0.637	0.001
*PRKCQ*	0.477	0.021

^1^ *r* is correlation coefficient; ^2^ *p*-values remained statistically significant after FDR correction.

## Data Availability

Not applicable.

## Supplementary Materials

The following are available online at https://www.mdpi.com/article/10.3390/nu13072313/s1, Table S1: Table showing the correlation of changes in gene expression before and after combined exercise and nutrient supplement intervention.

## References

[1] Larsson L., Degens H., Li M., Salviati L., Lee Y.I., Thompson W., Kirkland J.L., Sandri M. (2019). Sarcopenia: Aging-Related Loss of Muscle Mass and Function. Physiol. Rev..

[2] Makanae Y., Fujita S. (2015). Role of Exercise and Nutrition in the Prevention of Sarcopenia. J. Nutr. Sci. Vitaminol..

[3] Yoshimura Y., Wakabayashi H., Yamada M., Kim H., Harada A., Arai H. (2017). Interventions for Treating Sarcopenia: A Systematic Review and Meta-Analysis of Randomized Controlled Studies. J. Am. Med. Dir. Assoc..

[4] Wu H., Xia Y., Jiang J., Du H., Guo X., Liu X., Li C., Huang G., Niu K. (2015). Effect of Beta-Hydroxy-Beta-Methylbutyrate Supplementation on Muscle Loss in Older Adults: A Systematic Review and Meta-Analysis. Arch. Gerontol. Geriatr..

[5] Cruz-Jentoft A.J., Landi F., Schneider S.M., Zúñiga C., Arai H., Boirie Y., Chen L.-K., Fielding R.A., Martin F.C., Michel J.-P. (2014). Prevalence of and Interventions for Sarcopenia in Ageing Adults: A Systematic Review. Report of the International Sarcopenia Initiative (EWGSOP and IWGS). Age Ageing.

[6] Beckwée D., Delaere A., Aelbrecht S., Baert V., Beaudart C., Bruyere O., de Saint-Hubert M., Bautmans I. (2019). Exercise Interventions for the Prevention and Treatment of Sarcopenia. A Systematic Umbrella Review. J. Nutr. Health Aging.

[7] Beaudart C., Dawson A., Shaw S.C., Harvey N.C., Kanis J.A., Binkley N., Reginster J.Y., Chapurlat R., Chan D.C., Bruyère O. (2017). Nutrition and Physical Activity in the Prevention and Treatment of Sarcopenia: Systematic Review. Osteoporos. Int..

[8] Denison H.J., Cooper C., Sayer A.A., Robinson S.M. (2015). Prevention and Optimal Management of Sarcopenia: A Review of Combined Exercise and Nutrition Interventions to Improve Muscle Outcomes in Older People. Clin. Interv. Aging.

[9] Englund D.A., Kirn D.R., Koochek A., Zhu H., Travison T.G., Reid K.F., von Berens Å., Melin M., Cederholm T., Gustafsson T. (2017). Nutritional Supplementation With Physical Activity Improves Muscle Composition in Mobility-Limited Older Adults, The VIVE2 Study: A Randomized, Double-Blind, Placebo-Controlled Trial. J. Gerontol. Ser. A.

[10] Cruz-Jentoft A.J. Beta-Hydroxy-Beta-Methyl Butyrate (HMB): From Experimental Data to Clinical Evidence in Sarcopenia. http://www.eurekaselect.com/152747/article.

[11] Deutz N.E.P., Pereira S.L., Hays N.P., Oliver J.S., Edens N.K., Evans C.M., Wolfe R.R. (2013). Effect of β-Hydroxy-β-Methylbutyrate (HMB) on Lean Body Mass during 10 Days of Bed Rest in Older Adults. Clin. Nutr..

[12] Oktaviana J., Zanker J., Vogrin S., Duque G. (2019). The Effect of β-Hydroxy-β-Methylbutyrate (HMB) on Sarcopenia and Functional Frailty in Older Persons: A Systematic Review. J. Nutr. Health Aging.

[13] Din U.S.U., Brook M.S., Selby A., Quinlan J., Boereboom C., Abdullah H., Franchi M., Narici M.V., Phillips B.E., Williams J.W. (2019). A Double-Blind Placebo Controlled Trial into the Impacts of HMB Supplementation and Exercise on Free-Living Muscle Protein Synthesis, Muscle Mass and Function, in Older Adults. Clin. Nutr..

[14] Silva V.R., Belozo F.L., Micheletti T.O., Conrado M., Stout J.R., Pimentel G.D., Gonzalez A.M. (2017). β-Hydroxy-β-Methylbutyrate Free Acid Supplementation May Improve Recovery and Muscle Adaptations after Resistance Training: A Systematic Review. Nutr. Res..

[15] Skaaby T., Thuesen B.H., Linneberg A. (2018). Vitamin D, Sarcopenia and Aging. Vitam. D Clin. Med..

[16] Remelli F., Vitali A., Zurlo A., Volpato S. (2019). Vitamin D Deficiency and Sarcopenia in Older Persons. Nutrients.

[17] Bauer J., Biolo G., Cederholm T., Cesari M., Cruz-Jentoft A.J., Morley J.E., Phillips S., Sieber C., Stehle P., Teta D. (2013). Evidence-Based Recommendations for Optimal Dietary Protein Intake in Older People: A Position Paper from the PROT-AGE Study Group. J. Am. Med. Dir. Assoc..

[18] Ross A.C. (2011). The 2011 Report on Dietary Reference Intakes for Calcium and Vitamin D. Public Health Nutr..

[19] Logan S.L., Spriet L.L. (2015). Omega-3 Fatty Acid Supplementation for 12 Weeks Increases Resting and Exercise Metabolic Rate in Healthy Community-Dwelling Older Females. PLoS ONE.

[20] Deyhle M.R., Hyldahl R.D. (2018). The Role of T Lymphocytes in Skeletal Muscle Repair From Traumatic and Contraction-Induced Injury. Front. Physiol..

[21] Livshits G., Kalinkovich A. (2019). Inflammaging as a Common Ground for the Development and Maintenance of Sarcopenia, Obesity, Cardiomyopathy and Dysbiosis. Ageing Res. Rev..

[22] Min J.L., Barrett A., Watts T., Pettersson F.H., Lockstone H.E., Lindgren C.M., Taylor J.M., Allen M., Zondervan K.T., McCarthy M.I. (2010). Variability of Gene Expression Profiles in Human Blood and Lymphoblastoid Cell Lines. BMC Genom..

[23] Zhu L.-Y., Chan R., Kwok T., Cheng K.C.-C., Ha A., Woo J. (2019). Effects of Exercise and Nutrition Supplementation in Community-Dwelling Older Chinese People with Sarcopenia: A Randomized Controlled Trial. Age Ageing.

[24] Chen L.-K., Liu L.-K., Woo J., Assantachai P., Auyeung T.-W., Bahyah K.S., Chou M.-Y., Chen L.-Y., Hsu P.-S., Krairit O. (2014). Sarcopenia in Asia: Consensus Report of the Asian Working Group for Sarcopenia. J. Am. Med. Dir. Assoc..

[25] Vandesompele J., De Preter K., Pattyn F., Poppe B., Van Roy N., De Paepe A., Speleman F. (2002). Accurate Normalization of Real-Time Quantitative RT-PCR Data by Geometric Averaging of Multiple Internal Control Genes. Genome Biol..

[26] Benjamini Y., Hochberg Y. (1995). Controlling the False Discovery Rate: A Practical and Powerful Approach to Multiple Testing. J. R. Stat. Soc. Ser. B (Methodol.).

[27] McCormick R., Vasilaki A. (2018). Age-Related Changes in Skeletal Muscle: Changes to Life-Style as a Therapy. Biogerontology.

[28] Carlson L.A., Tighe S.W., Kenefick R.W., Dragon J., Westcott N.W., LeClair R.J. (2011). Changes in Transcriptional Output of Human Peripheral Blood Mononuclear Cells Following Resistance Exercise. Eur. J. Appl. Physiol..

[29] Rong Y.-D., Bian A.-L., Hu H.-Y., Ma Y., Zhou X.-Z. (2018). Study on Relationship between Elderly Sarcopenia and Inflammatory Cytokine IL-6, Anti-Inflammatory Cytokine IL-10. BMC Geriatr..

[30] Huang S.-W., Xu T., Zhang C.-T., Zhou H.-L. (2020). Relationship of Peripheral Lymphocyte Subsets and Skeletal Muscle Mass Index in Sarcopenia: A Cross-Sectional Study. J. Nutr. Health Aging.

[31] Ogawa S., Yakabe M., Akishita M. (2016). Age-Related Sarcopenia and Its Pathophysiological Bases. Inflamm. Regen..

[32] Zhang J., Xiao Z., Qu C., Cui W., Wang X., Du J. (2014). CD8 T Cells Are Involved in Skeletal Muscle Regeneration through Facilitating MCP-1 Secretion and Gr1(High) Macrophage Infiltration. J. Immunol..

[33] Castiglioni A., Corna G., Rigamonti E., Basso V., Vezzoli M., Monno A., Almada A.E., Mondino A., Wagers A.J., Manfredi A.A. (2015). FOXP3+ T Cells Recruited to Sites of Sterile Skeletal Muscle Injury Regulate the Fate of Satellite Cells and Guide Effective Tissue Regeneration. PLoS ONE.

[34] Fuller D.M., Zhu M., Song X., Ou-Yang C., Sullivan S.A., Stone J.C., Zhang W. (2012). Regulation of RasGRP1 Function in T Cell Development and Activation by Its Unique Tail Domain. PLoS ONE.

[35] Steinke F.C., Xue H.-H. (2014). From Inception to Output, Tcf1 and Lef1 Safeguard Development of T Cells and Innate Immune Cells. Immunol. Res..

[36] Cornely R., Pollock A.H., Rentero C., Norris S.E., Alvarez-Guaita A., Grewal T., Mitchell T., Enrich C., Moss S.E., Parton R.G. (2016). Annexin A6 Regulates Interleukin-2-Mediated T-Cell Proliferation. Immunol. Cell Biol..

[37] Barata J.T., Durum S.K., Seddon B. (2019). Flip the Coin: IL-7 and IL-7R in Health and Disease. Nat. Immunol..

[38] Isakov N., Altman A. (2012). PKC-Theta-Mediated Signal Delivery from the TCR/CD28 Surface Receptors. Front. Immunol..

[39] Demonbreun A.R., Fallon K.S., Oosterbaan C.C., Bogdanovic E., Warner J.L., Sell J.J., Page P.G., Quattrocelli M., Barefield D.Y., McNally E.M. (2019). Recombinant Annexin A6 Promotes Membrane Repair and Protects against Muscle Injury. J. Clin. Investig..

[40] Butler M.H., David C., Ochoa G.C., Freyberg Z., Daniell L., Grabs D., Cremona O., De Camilli P. (1997). Amphiphysin II (SH3P9; BIN1), a Member of the Amphiphysin/Rvs Family, Is Concentrated in the Cortical Cytomatrix of Axon Initial Segments and Nodes of Ranvier in Brain and around T Tubules in Skeletal Muscle. J. Cell Biol..

[41] Harries L.W., Hernandez D., Henley W., Wood A.R., Holly A.C., Bradley-Smith R.M., Yaghootkar H., Dutta A., Murray A., Frayling T.M. (2011). Human Aging Is Characterized by Focused Changes in Gene Expression and Deregulation of Alternative Splicing. Aging Cell.

