# Supplementary material for: Peripheral Blood T Cell Gene Expression Responses to Exercise and HMB in Sarcopenia
Source: Nutrients. 2021 Jul 5;13(7):2313. doi: 10.3390/nu13072313 (PMC8308783; doi:10.3390/nu13072313)
Supplement: Supplementary file 1 [file nutrients-13-02313-s001.zip › nutrients-1250034-supplementary.pdf]

Supplementary Table S1. Table showing the correlation of changes gene expression before and after combined exercise and nutrient supplement intervention.

Gene	Fold Change	*p*-Value
GIMAP7	1.44	0.180
PRKCQ	3.01	**0.020**
LRRN3	1.71	0.170
NELL2	1.56	0.120
ANXA6	1.75	**0.023**
IL32	1.49	0.170
IL7R	1.88	0.086
RASGRP1	1.66	0.110
BIN1	2.76	**0.035**
CD3D	1.22	0.450
GOLGA8A	1.05	0.092
LCK	1.31	0.160
MIF	1.05	0.920
CD6	1.26	0.250
GZMK	1.06	0.340
ITGA6	1.19	0.540
CTSW	1.93	0.310
EOMES	2.79	0.390
PRKCA	3.35	0.430
HNRNPA1	1.01	0.800
CD8A	2.70	0.870
CD5	2.05	0.820
CCR7	2.05	0.990
MAF	1.36	**0.016**
TCF7	1.76	0.690
HINT1	1.07	**0.008**
CCT2	1.18	0.290
KLRK1	2.08	0.800
TOMM7	0.97	**0.014**
CD69	1.23	0.170
EIF3E	0.87	**0.004**
SOD1	1.05	**0.004**
LDHB	1.20	**0.008**
LEF1	1.80	0.340
CCL5	1.22	0.075
CD27	1.68	0.390
PRKCQAS1	1.46	0.450
BCL2	1.46	0.520

p-value in bold are genes showing significant p-value.
